# Antiviral Properties of Human Milk

**DOI:** 10.3390/microorganisms9040715

**Published:** 2021-03-31

**Authors:** Sophie I. S. Wedekind, Natalie S. Shenker

**Affiliations:** 1 Department of Surgery and Cancer, Imperial College London, London W12 0NN, UK; s.wedekind@imperial.ac.uk; 2 Human Milk Foundation, Daniel Hall Building, Rothamsted Institute, Harpenden AL5 2JQ, UK

**Keywords:** lactation, global health, therapeutics, viruses

## Abstract

Humans have always coexisted with viruses, with both positive and negative consequences. Evolutionary pressure on mammals has selected intrinsic properties of lactation and milk to support the relatively immunocompromised neonate from environmental pathogens, as well as support the normal development of diverse immune responses. Human milk supports both adaptive and innate immunity, with specific constituents that drive immune learning and maturation, and direct protection against microorganisms. Viruses constitute one of the most ancient pressures on human evolution, and yet there is a lack of awareness by both public and healthcare professionals of the complexity of human milk as an adaptive response beyond the production of maternal antibodies. This review identifies and describes the specific antiviral properties of human milk and describes how maternal support of infants through lactation is protective beyond antibodies.

## 1. Introduction

Human milk is a complex biofluid, containing not just tailored nutrition to satisfy growth requirements, but developmental cues and protection of the relatively immunocompromised infant against pathogens. As an evolutionary strategy, lactation enables the infant to transition from placental protection to an environment full of microbes. With minimally developed adaptive immune systems, infants rely on an immature innate immune system in a predominant Th2 state as the first line of their defence [1,2]. Human milk contains various mechanisms and bioactive molecules that compensate for these limitations and support normal immune development throughout a natural term of lactation up to several years postnatally. Milk contains an abundance of antimicrobial and immunomodulatory molecules which aid in antiviral defence to aid the immature infant immune system [3,4].

Along with the transfer of maternal antibodies, other immunological and bioactive activity is provided via human milk. Cellular immunity is provided by macrophages and leukocytes, which are highly abundant at the beginning of lactation and particularly in colostrum–the specialised fluid produced for several days postnatally before the onset of the second stage of lactogenesis [5]. Multiple maternal characteristics influence the cellular composition of human milk, such as maternal age and certain aspects of diet [6,7]. However, a lack of studies makes it difficult to elucidate the effects these changes in composition have on the infant and their health, both in the short and long term.

**Figure 1:**
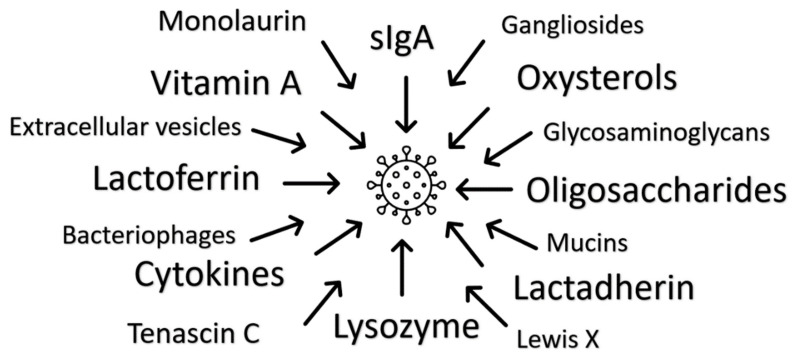
Multiple antiviral mechanisms of human milk have been identified. There is presumed individual and additional multiplicative functionality, with redundancy indicating the significance of milk’s immune function.

In this review, the panoply of antiviral properties of human milk components are explored in relation to their antiviral properties with a view to highlighting novel areas of research and therapeutic potential Figure 1. Undoubtedly, human milk is most effective when infants are fed directly with their mother’s milk, but human milk banks may be able to direct batches of donated milk with specific properties to infants with specific clinical need. It remains unclear whether the adaptation of infant formula with single agents modelled from human milk function will add any benefit.

## 2. Immunoglobulins

**Figure 2:**
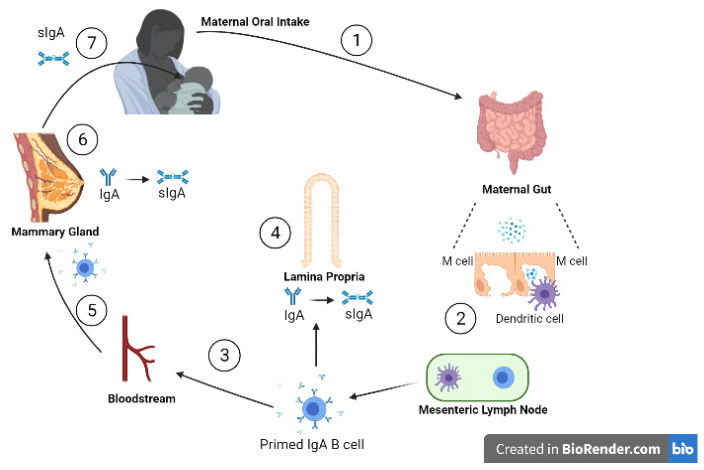
Entero-mammary circulation allows the transportation of maternal immunoglobulins into the infant, via human milk. (**1**) Viral particles are ingested orally and pass to the gut; (**2**) inside the gut wall dendritic cells sample viral antigens, presenting them to local lymphatic cells; (**3**) primed IgA B cells travel to (**4**) the lamina propria and (**5**) the mammary stroma lining the ductal system via the bloodstream; (**6**) on the basolateral side of the mammary ductal system, IgAs are produced by plasma cells in the mammary gland. The IgAs are transported across alveolar epithelial cells by polymeric Ig receptors, where they bind to the secretory components to form sIgAs; (**7**) sIgA passes into the infant gut lumen to provide protection from viruses through a range of mechanisms. (adapted from Newburg D and Walker W, 2007 [12]). Created with BioRender.com.

Approximately 90% of the immunoglobulins in human milk are IgA while 8% is IgM, with the remainder made up of IgG [8]. Secretory immunoglobulin A (sIgA), IgAs are relatively stabilised from proteolytic action by binding to the secretory component (SC), and is the most predominant form of the immunoglobulins present in human milk [9]. sIgAs can neutralise invading viruses directly by binding and mediating noninflammatory extracellular and intracellular immune exclusion by inhibiting their adherence to the mucosal epithelia [10,11]. The transfer of sIgA, shown in Figure 2, begins when the mother is exposed to an enteric pathogen. This pathogen is presented to maternal dendritic cells activating T lymphocytes and ultimately stimulating plasma cells to produce IgA on the basolateral side of the mammary epithelial cell. IgA then traverses the mammary cell to enter the milk as sIgA [12].

As sIgA is resistant to proteolysis, it is able to reach the infant’s gut lumen [13,14]. However, in preterm infants at least, non-sIgA levels decreased as a result of protease activity, independent of gastric acid which is not produced at that stage, whereas total sIgA, IgM and IgG survived mostly intact [15]. This study concluded that the longer the human milk immunoglobulins are able to remain in the body of the infant, the longer they can provide passive immunity while the immune system develops.

Vaccine studies give a unique clinical perspective on antibody transfer via milk. One study observed Bangladeshi mothers who received either trivalent inactivated influenza vaccine, or 23-valent pneumococcal polysaccharide vaccine during their third trimester of pregnancy. They found higher levels of viral neutralisation activity in vaccinees, which correlated with influenza-specific IgA levels and significantly decreased the expected number of respiratory illnesses with fever episodes in infants with influenza-vaccinated mothers [16]. A more recent study investigated SARS-CoV-2-specfic IgA from milk of women who had recovered from COVID-19 [17]. Of the samples collected, 80% showed significant IgA binding activity to the receptor binding domain and that out of measuring IgA, IgM and IgG, IgA was the most dominant response. This was supported by the report by Pace et al., which confirmed neutralising capacity for anti-SARS-CoV-2-specfic IgA and IgG in human milk, suggesting milk-derived immunoglobulins could be a possible therapeutic intervention for COVID-19 [18]. However, human milk immunoglobulins may have limited antimicrobial function in isolation, suggesting that other factors within human milk contribute to the overall antiviral activity in antibody-positive human milk [19,20,21]. Further research needs to understand the extent of milk-derived immunoglobulins antiviral activity and how interactions with the other various components in human milk can impact their overall activity.

## 3. Human Milk Oligosaccharides (HMOs)

As the third largest class of components within human milk after lactose and lipids, HMOs are a family of free oligosaccharides which are diverse in structure and unique to human milk [8,22]. HMO composition varies between women and over the course of the lactation period, as well as by season of the year [23]. The concentration of HMOs in early milk can be as much as 20–25 g/L, and is thought to decline as the milk matures, although scant research on full lactation durations (beyond 2 years) exists [24,25]. Lactose serves as the template for all HMOs at the reducing end and can be elongated, fucosylated or sialylated, causing variations which creates over a hundred different HMOs [26].

**Figure 3:**
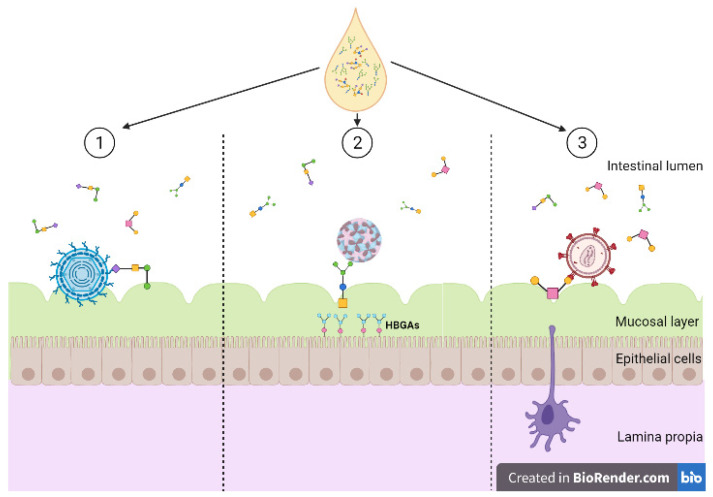
Diversity of human milk oligosaccharide (HMO) interference against viral entry. (**1**) HMOs act as soluble decoy receptors for rotavirus, preventing direct binding to host cells; (**2**) HMOs mimic histo-blood group antigens (HBGAs), binding to both GI and GII HBGA pockets; (**3**) HMOs bind to glycoprotein gp120 interfering and preventing viral binding to dendritic cell-SIGN. Created with BioRender.com.

Of the multiple functions of HMOs, immunomodulation is one of the most significant [27,28]. Viruses invade and colonise by binding to host cells, utilising cell surface glycoconjugates as receptors, inducing oxidative stress and anti-inflammatory signals [29]. Some HMOs express glycans that can bind onto surface lectins on the host cells which prevents viral molecules binding and invading these cells, acting as receptor decoys Figure 3 [30]. HMOs may indirectly reduce immune responses by altering the composition of the infant gut microbiota [31]. A study which investigated this looked at infant formula supplemented with 2′FL (1 g/L) and LNnT (0.5 g/L) compared to those fed without supplemented formula [32]. Those that were supplemented had a significantly different microbiota composition in comparison, where Bifidobacterium was more abundant. However, this study focussed on formula fed infants rather than exclusively breastfed infants, and therefore it is not able to show the difference in effect between formula supplemented and exclusively breastfed. A number of studies indicated the relationship between the gut microbiota composition and inflammation [33,34]. While there is evidence supporting HMOs indirect changes to aid in the infant’s immune response, there are also results from in vitro studies which show their ability to directly modulate the immune response [35].

Specific research has identified the role of HMOs in preventing infection with several viruses across different viral classes. For example, rotaviruses constitute the most common cause of diarrhoea in infants and children worldwide and is the main agent towards gastroenteritis [36]. Prevention can be via vaccination, but this is not indicated before the first two months postnatally. Viral entry is a complex multi-step process where different rotavirus surface proteins interact with different cell surface receptors [37]. Viral proteins VP7 and VP4 are involved with the receptor binding and permeabilise membranes [29,38]. Rotavirus entry may occur via calcium-dependent endocytosis, where an observed decrease in calcium concentration solubilises the surface protein from the virus [39]. HMOs have been observed to directly intervene with rotavirus acting as soluble decoy receptors for the rotavirus reducing infectivity [40]. Specific HMOs were shown to reduce infectivity of human rotavirus strains, as well as impacts on colonic microbiota and cytokine responses. However, it was noted that there were differences in effect between rotavirus strains, but research is lacking on the effects of different HMO concentrations against different rotavirus species [41].

Noroviruses are small non-enveloped RNA viruses. Susceptibility to norovirus infection depends on a genetic trait caused by the expression of histo-blood group antigens (HBGAs) [42]. The host mucosal surface in the gastrointestinal tract contains HBGAs that facilitate the binding of noroviruses [43,44]. HGBAs are carbohydrate-based antigens which include the Lewis and ABH antigens expressed on the cell surface of red blood cells and mucosal surface epithelial cells. Binding is strain-dependent and occurs by the viral capsid protein via the P domain [43]. X-ray crystallography has shown that HMOs interact with norovirus by mimicking HBGAs [45]. Specifically, 2′-fucosyllactose (2′FL) HMOs able to block multiple strains of norovirus, by being able to bind to both GI and GII HBGA pockets. An investigation focussing on high-molecular mass HMOs and their binding ability with a specific strain of norovirus (GII.4, Sydney, Australia, 2012, JX459908) showed these types of HMOs have a higher affinity for binding compared to monovalent HMOs as a result of the higher avidity of α-fucose [46]. While these studies have highlighted the ability of certain HMOs to bind sufficiently to norovirus strains, most HMOs are bound to a lipid or protein carrier rather than present as free oligosaccharides, such as 2′FL or 3FL. There is a need for more research to understand the efficacy of HMOs in their various forms against multiple strains of noroviruses.

Human immunodeficiency virus (HIV) comprises two species of the Lentivirus family, named HIV-1 and HIV-2. These are single-stranded enveloped RNA viruses which integrate with the host cellular DNA. HIV can be latent for 2 to 10 years and avoids detection from the immune system; once active the pre-virus DNA is transcribed into RNA and ultimately leads to the expression of new virus particles which are readily able to attack CD4 T lymphocytes [40]. Eventually this leads to the individual being at high risk of diseases and other infection [47]. With modern anti-retroviral therapy, the risk of transmission for a mother with undetectable viral load if the infant is exclusively human milk fed is low (<0.5%). However, if the mother has detectable levels of virus during pregnancy or postnatally, HIV-1 can be transmitted from the mother to infant via breastfeeding only around 10–15% of infants become ill from an HIV-infected mother when breastfed exclusively [48]. Interestingly, if the infant is mixed fed and receives some infant formula alongside breastfeeding, the risk again rises to 6–10%, suggesting even small doses of formula override the protective effects of breastfeeding.

HIV viruses interact with the cell surface receptor dendritic cell-specific intercellular adhesion molecule-3-grabbing non-integrin (DC-SIGN) to enter the cell [49]. These receptors are on the surface of macrophages and dendritic cells, allowing HIV to self-replicate and transform CD4+ T lymphocytes [50]. HMOs isolated in an ELISA assay show prevention of glycoprotein gp120 on the surface of HIV-1 from binding on the DC-SIGN [51]. HIV-infected mothers with HMOs above the median average concentration were less likely to transmit HIV via breastfeeding [8,52]. Higher concentrations of 3′-sialyllactose (3-SL) HMOs were associated with protection against postnatal HIV transmission, but this type of HMO is significantly higher in HIV-infected women compared to uninfected women [8]. A follow-up study investigated the differences in HMO profiles between HIV-infected and HIV-uninfected mothers, confirming higher quantities of 3′-SL in HIV-infected mothers [52]. The authors proposed that the difference could be due to HIV infection changing the glycosylation process in the mammary epithelial gland epithelial cell and therefore changing the composition of HMOs, or that HIV-infected individuals differentially express glycosylation-related genes, either greater or fewer.

## 4. Lactoferrin (LF)

LF is abundant in the colostrum, with a concentration up to 7 g/L with a decline over the initial few months of lactation [53]. Perrin et al. showed that the concentration of LF then increases as lactation continues into the second year [54]. LF is multifactional iron binding protein that leads to the inhibition of microbial growth but independent of the iron binding site. LF also interacts with microbial, viral and cell surfaces, preventing adhesion and entry of pathogens into the host cells [55]. LF receptors have been identified on the surface of different immune cells, such as macrophages, lymphocytes and dendritic cells [56,57]. Multiple viruses are inhibited by LF, most likely by preventing infection of the host cell rather than inhibiting viral replication [58]. LF either binds directly to viral particles or by binding to the host cell macromolecules, preventing the virus to bind on to the cell surface receptors, depending on the viruses. It is suggested that LF’s wide range of activities may be due to its capacity to bind to iron and its ability to interfere with both the virus and host cellular receptors to prevent attachment [59].

Herpesviridae viruses, including herpes simplex virus (HSV), part of the alpha-herpes virus family, and cytomegalovirus (CMV), part of the beta-herpes virus family, are both prevented from entering the target cell by LF [60,61]. Multiple groups have confirmed LFs antiviral activity against HSV-1 and -2 in vitro during early phase infections [62,63]. Hammer et al. demonstrated that fragments of the protein from the N-lobe presented antiviral activity, but the full protein proved to be more potent in effect [62]. For CMV, the N-terminal region of LF has been demonstrated to be an essential part for its antiviral activity [64]. A more recent study demonstrated LF’s ability to neutralise CMV in vitro using milk from mothers of preterm infants and their infant’s saliva [65]. However, the concentrations of LF in human milk and salivary samples were deemed likely too low for effective neutralisation in vivo, but this should be explored in future studies.

While LF can directly interfere with viral activity, receptors are also a crucial component to further aid this effect. Heparan sulphate proteoglycans (HSPGs) are central to the ability of many viruses to dock onto target cells [66,67]. LFs inhibit this action by binding to these receptors, inhibiting cell entry by several viruses, including SARS-CoV [68]. LF blocks the infection of cells by SARS-CoV by directly binding to HSPG [69]; however, no evidence yet exists as to whether this is also true for SARS-CoV-2.

LF may further induce indirect antiviral activity via upregulation of the immune system. In vitro cell culture and trials in human volunteers demonstrated that orally administered LF upregulated NK-cells and macrophages [70]. However, a randomised placebo-controlled trial examined external LF supplementation in preterm infants, finding it did not prevent late-onset infection [71]. However, this study used lactoferrin mixes with a range of fluids, encompassing sterile water, human milk and formula, which may have impacted their findings. It is likely that LF interacts with numerous other components of human milk to mediate its range of antiviral and other effects. Additionally, collected data in a meta-analysis indicate that bovine LF supplementation does not provide convincing evidence to be used as a therapeutic option to reduce late-onset neonatal sepsis, which may be due to structural differences between human and bovine LF [72,73].

## 5. Lysozyme

Lysozyme is an enzyme found in most biological secretions and leukocytes which acts as an antioxidant. Lysosomes, along with lactoferrins, are some of the most abundant antimicrobials and are widely distributed in animal secretions and tissues, such as human milk and tear fluid [74]. Lysozymes have also been observed to have anti-inflammatory action in which a study demonstrated to inhibit haemolytic activity of serum complement where the activity was dose-dependent when comparing milk samples with complement activation and normal human milk samples [75]. A few studies have investigated the effect lysozymes have on HIV-1, with one using lysozymes contained in egg whites showing HIV-1 attachment inhibition to host cell CD4 [76]. Another looked at human lysozymes and demonstrated anti-HIV activity is associated with the beta-core fraction of human chorionic gonadotropin [77].

Lysozyme content in milk was observed to increase as the milk matured from colostrum to mature milk and continue into the second year of lactation [54,78]. However, a 2001 study showed lysozyme concentration decreased from colostrum to transitional milk but increased after day 29 within mature milk where highest lysozyme content was present in day 57–84 [79]. The variability of lysozyme concentration has yet to be investigated in relation to its effect on sufficient antiviral activity.

## 6. Lactadherin

Lactadherin is a 46 kDa mucin-associated glycoprotein [80]. Lactadherin can inactivate virus’s infectivity by increasing phagocytosis of apoptotic cells, via the sialic acid component directly interacting with the virus [81]. This was demonstrated in studies using rotavirus, where lactadherin binds specifically to all four strains which inhibits their ability to bind to host cell receptors and limit inflammation [82]. Concentrations of lactadherin vary between mothers [80]. However, despite the difference, the protein showed a significant association with symptoms in rotavirus-infected infants that were breastfed [80]. A more recent study looked at lactadherin in rats and comparing its ability to prevent necrotizing enterocolitis (NEC) [83]. They demonstrated oral administration of lactadherin improves the prevention of NEC within the rat models, perhaps due to the accelerated maturation of the intestine, anchoring specific tight junction proteins, claudin 3, occludin, and E-cadherin, to protect the intestinal barrier. However, this has not been confirmed by other studies looking into human milk, in vitro or vivo. There is little recent evidence looking into the antiviral effect of human lactadherin beyond rotavirus and its influence within infants.

## 7. Tenascin C

Tenascin C (TNC) is an extracellular matrix protein which is important in development and wound healing [84]. TNCs antiviral properties have been demonstrated by its ability to neutralise HIV-1 [85,86]. A 2013 study elucidated purified TNC captures and mediates the neutralisation of HIV-1 variants by binding to the HIV-1 Envelope protein chemokine coreceptor site that would otherwise bind to its primary receptor CD4 [85]. It was noted that TNC may not be present in sufficient amounts in human milk or other mucosal fluid to mediate effective HIV-1 neutralisation in vivo, although perhaps due to the presence of other antiviral components present in human milk, including those mentioned in this review, may, overall, provide neutralisation of HIV-1, but this needs to be investigated [86]. Although, a fairly recent study has elucidated the strategies of TNC neutralisation of HIV-1 within mucosal fluids, where TNC binding and neutralisation relies on its fibrinogen-like glove and fibronectin-type III domains along with mediated neutralisation dependent on specific Env V3 residues [87]. It is also worth noting that the difference in concentration of TNC has not been investigated between mothers and whether these differences may have varying impacts towards antiviral activity.

## 8. Extracellular Vesicles

Extracellular vesicles (EV) are submicron sized vehicles released by cells for intercellular communication, they are packed with active regulatory and stimulatory molecules from the parent cell, including miRNAs [88,89]. In human milk-derived EV, a total of 1963 proteins have been identified, with a sizable overlap between different donors [90]. EVs were detected to have MHC classes I and II, CD63, CD81 and CD86 on the vesicles as well as vesicle preparation inhibited anti-CD3-induced IL-1 and IFN-γ production [91]. Admyre and colleagues further found that breast tissue contributes to the human milk-derived EV pool and immune cells are likely to produce milk-derived changes in the EV proteome over the lactation period.

## 9. Bacteriophages

Bacteriophages are bacterial viruses, in which bacteriophage lytic enzymes (lysins) weaken the bacterial cell wall. They have been detected in low levels within infants shortly after birth but are thought to have maternal and environmental origins [92]. It has been found that within the first 2 weeks of life, the phage communities undergo dramatic changes in diversity and abundance within the infant gut [93]. Infant and mother pairs showed human milk may be important as an initial source of phages within the infant gut when characterising viromes [94,95]. In the gut bacteriophages are estimated to be as abundant as their bacterial hosts and provide a source of carbohydrate metabolism genes and antibiotic resistance along with providing cofactors which promote bacterial growth and development [96]. A recent study demonstrated that viral colonisation in early life is stepwise, where the first step is characterised by the induction of prophages from pioneer bacteria [97]. The second step is characterised by the colonisation of viruses which infect human cells, which is regulated by breastfeeding.

It is suggested that the phages interact with circulating dendritic cells and macrophages in the lymphatic system where direct stimulation of immune responses occur to generate pro- or anti-inflammatory responses and create specific antiphage-neutralising antibodies [92]. Dysbiosis of phage communities has been associated with various inflammatory diseases, such as type 2 diabetes and inflammatory bowel syndrome [98,99].

## 10. Cytokines

Cytokines are multifunctional glycoproteins which are involved in immune activation and cell communication [100]. They operate in networks, producing a cascade of effects which contribute to the development and functions of the immune system, including mediating and regulating inflammatory responses [101]. The discovery of cytokine activities was the first indication of cytokines being present in human milk, including growth, differentiation, and production of immunoglobulins by B cells [102].

Transforming growth factor beta (TGF-β) is of the most abundant cytokines in human milk and is converted in its active form by the low pH of the stomach [103,104]. Milk-borne TGF-β regulates inflammation and aids in preventing allergic diseases and inhibiting pro-inflammatory cytokines [100]. TGF-β suppresses the transcription of IFN-γ by inhibiting its transcriptional regulator [105]. TGF-β of human milk has been shown to modulate MHC class II molecule expression on intestinal epithelial cells, which may contribute to the infant’s ability to cope with antigen presence after birth [106]. Infants with necrotizing enterocolitis (NEC) have been shown to have low levels of mucosal TGF-β expression and reduced TGF-β bioactivity [107]. An animal study investigating preterm pigs with NEC that were fed porcine milk showed a decrease in the severity of NEC by distinguishing the difference of elevated intestinal weight, mucosa proportion and villus height [108]. The promotion of villus growth us due to TGF-β phosphorylating its receptor and increases the expression of tight junction proteins which are vital in epithelia permeability regulation [105,109]. This suggests that TGF-β has multiple roles, such as tissue repair and regulating the cellular immune response and cytokine profile, which aid in the maturation of the infant’s immune system.

Variability of cytokine levels was observed between individual women and women delivering preterm infants compared to women delivering full term infants, where lower levels of multiple cytokines was found in the colostrum of mothers with preterm infants [110]. Though, cytokine levels remained high during the lactation period which spanned from months to years. Further research is needed to identify and further understand the other various cytokines present in human milk and the capacity of their antiviral protection.

## 11. Glycosaminoglycans

Glycosaminoglycans (GAGs) are mucopolysaccharides that are grouped generally into four classes: hyaluronan, keratan sulphate, galactosaminoglycans (represented by chondroitin sulphate) and dermatan sulphate, all which are present in human milk [111,112]. Their various molecular structures contribute several biological roles, including cell growth, cell differentiation and cell to cell matrix interactions [113]. Coppa et al. found the highest GAG values within day 4 of lactation but this number showed a strong decrease between days 4 to 10 in both term and preterm infants [114]. Their antiviral ability was demonstrated with its interaction with HIV. Newburg et al., found the multiple forms of GAGs inhibited the ability for the HIV envelope glycoprotein, gp120, to bind to the host cell receptor, CD4, which a vital first step for HIV infectivity [115]. It was further identified that this effect by GAGs were uncompromised by digestion with lytic enzymes, specifically for heparin, heparan sulphate and dermatan sulphate. It is also thought that GAGs could contribute to the antioxidant effect of human milk and have shown to induce the activation of antioxidant enzymes [116]. There has been a lack of studies looking into GAGs beyond 2013. Further studies are needed to elucidate the roles of the various GAGs and their effect towards antiviral activity.

## 12. Mucins

Mucins are the main gel-forming component which play an important role in protein secretion, protein stability and function. Human milk contains trans-membrane mucins, MUC1 and MUC4, with MUC1 the most widely distributed mucin and is expressed in the mammary gland [117,118,119]. MUC1 and MUC4 has been shown to inhibit HIV-1 activity by around 97%, however this was in purified human milk in an in vitro assay and found no inhibition for crude human milk [120,121]. Although, Kazmi et al. demonstrated crude milk causes high levels of anti-HIV-1 activity comparable to saliva [122]. High levels of antiviral activity are associated with an affinity-purified complex of human milk mucin, and deglycosylation of the mucin complex led to the loss of antiviral activity [82]. The evidence so far indicates mucins have sufficient antiviral activity against HIV-1, but there is still a lack of recent evidence which further examines its efficacy, especially compared to saliva.

## 13. Lewis X

Lewis X (LeX) is a tetrasaccharide carbohydrate blood group antigen and has been demonstrated to play an important role in cell-to-cell recognition processes [123]. Studies have investigated the role of LeX and blocking the interaction between HIV-1 and CD4+ T cells. A study by Naarding et al. demonstrated this inhibitory effect that an LeX motif present in human milk can bind to DC-SIGN, expressed on dendritic cells [124]. Human milk from HIV-positive and -negative donors in Tanzania was analysed and indicated a correlation between HIV-inhibitory activity and the siaylated form of LeX in HIV-positive human milk [125]. The 2012 study also highlighted that despite this correlation, the same subjects had no inhibitory effect on cell-associated HIV infection in vitro. It is suggested that separate factors within human milk modulate cell-free versus cell-associated HIV-1 infection. Human milk protein MUC1 and its interaction with dendritic cell-specific intercellular adhesion molecule-3-grabbing non-integrin (DC-SIGN) showed to rely on LeX when binding to prevent pathogen interaction [126].

## 14. Monolaurin

Monolaurin is a monoester formed by lauric acid (medium-chain fatty acids). Monolaurin has been demonstrated to inactivate multiple viruses to some extent, including HIV, measles, Herpes simplex-1 and cytomegalovirus. An experiment using laboratory animals highlighted immune-boosting properties in lauric acid when feeding them coconut oil compared to those that were fed corn-oil [127]. There are no studies present that have examined the specific antiviral roles of monolaurin in human milk.

## 15. Vitamin A

Vitamin A is an important micronutrient for immunity, cellular differentiation, growth, vision and maintenance of epithelial surfaces. The relationship between vitamin A and immune function has been observed mainly in infants and children with vitamin A deficiency. A significant linear correlation was found between prenatal vitamin A levels and HIV-1 infected cells within mothers’ colostrum with a CD4 count under 400 [128]. Another study in Malawi found an inverse relationship between vitamin A levels and mother-to-child HIV 1 transmission risk, along with mothers with a vitamin A deficiency having a 3- to 4- fold increased risk of transmission. However, a study in Tanzania examined the effect of vitamin A and multivitamin (excluding vitamin A) supplementation on HIV-1 transmission, finding HIV-1 transmission increased [129]. It is unsure what aspect of the vitamin caused this effect, whether it was beta-carotene or vitamin A and its interference with retinoic acid [130]. A randomised placebo-controlled study postpartum is being conducted comparing vitamin A supplementation to understand the effect this has on infant immunity, in relation to dose and time point of postpartum supplementation, the results have yet to be published [131].

## 16. Gangliosides (GM1, GM2, GM3)

Gangliosides are molecules composed of glycosphingolipid and one or more sialic acids linked to a sugar chain. They act as antigens or receptors at the cell surface and have been shown to be responsible for mediating the intestinal immune system [132]. There are multiple forms of gangliosides, but the most abundant in human milk is GM. The concentrations of GM3 and GD3 were observed between colostrum and mature milk, and that over this period of lactation the most abundant was GD3 at the beginning to GM3 at the end of this period [133]. The antiviral properties of gangliosides have been demonstrated by examining its effect on bowel necrosis, where ganglioside preexposure suppressed infant bowel production of proinflammatory cytokines in response to LPS exposure and hypoxia [134].

## 17. Chondroitin Sulphate

Chondroitin sulphate (CS) is one of the most prominent class of GAG in human milk (55% of total GAG content) which has shown to be both an antiviral and antibacterial agent [111,135]. The CS has shown to stimulate specific antioxidant enzymes which could lead to inflammatory diseases [116]. Newburg and colleagues showed CS can inhibit the binding of HIV’s glycoprotein, gp120 to the host cell CD4 receptor [115]. This study is consistent with another which showed that CS in isolation can be protective to an intestinal in vitro system [136].

## 18. Oxysterols

Oxysterols are cholesterol oxidation derivatives. Viral infection induces the expression of CH25H in macrophages resulting in 25-hydroxyxholesterol (25OHC) production via enzymatic oxidation, which inhibits viral entry, assembly, and replication. This is likely due to the effects of the cholesterol content, as cholesterol is a vital part of the plasma membrane and intracellular lipid compartments. Studies have shown 25OHC and 27-hydroxycholesterol (27OHC) to have a wide range of antiviral activity [137,138,139]. Cagno and colleagues found the proinflammatory activity of the oxysterol and activation of NF-kB and IL-6 is produced by HSV-1 infection in vitro. Both 25OHC and 27OHC were found to upregulate IL-6 concentrations [138]. Another study highlighted that 25OHC and 27OHC affect the final step of viral penetration into the cells, and through this altering activity the recycling of cholesterol is disturbed [139]. This disturbance therefore results in an accumulation of cholesterol in the late endosomal compartment where the viral particles are sequestered and ultimately causes the prevention of viral replication. It was observed that 25OHC and 27OHC are present in milk from any stage of lactation, along with a report of 27OHC viral assays significantly blocking viral replication [140].

## 19. Conclusions

The therapeutic potential for human milk against viral disease is at this point almost entirely unexplored but may hold potential for treatments or preventative therapies against some of our most pressing pathogenic threats. Human milk antibodies have been found in over 80% of milk samples from women infected with COVID-19 [17], with data validated by our research group and others via personal communication (unpublished data). Further, the whey protein of human milk can inhibit viral infection and replication in SARS-CoV-2 infected cells [141]. In addition to the array of components described herein, -omic screens for human milk are in their earliest stages, and a full understanding of the individual and combinatorial anti-viral mechanisms of human milk is yet to be gained. A wider clinical and public understanding of the complexity of human milk components, mediating antiviral actions both individually and in combination, directly and indirectly, could enhance public health through both greater investment in the support of women who wish to breastfeed, and drive further research to unlock the therapeutic potential of human milk.

## Data Availability

Not applicable.

## References

[1] Ghazal P., Dickinson P., Smith C.L. (2013). Early life response to infection. Curr. Opin. Infect. Dis..

[2] Yu J.C., Khodadadi H., Malik A., Davidson B., Salles É.D.S.L., Bhatia J., Hale V.L., Baban B. (2018). Innate immunity of neonates and infants. Front. Immunol..

[3] Witkowska-Zimny M., Kaminska-El-Hassan E. (2017). Cells of human breast milk. Cell. Mol. Biol. Lett..

[4] Eriksen K.G., Christensen S.H., Lind M.V., Michaelsen K.F. (2018). Human milk composition and infant growth. Curr. Opin. Clin. Nutr. Metab..

[5] Hamosh M. (2001). Bioactive factors in human milk. Pediatr. Clin. N. Am..

[6] Lubetzky R., Sever O., Mimouni F.B., Mandel D. (2015). Human milk macronutrients content: Effect of advanced maternal age. Breastfeed. Med..

[7] Keikha M., Bahreynian M., Saleki M., Kelishadi R. (2017). Macro-and micronutrients of human milk composition: Are they related to maternal diet? A comprehensive systematic review. Breastfeed. Med..

[8] Bode L., Kuhn L., Kim H.Y., Hsiao L., Nissan C., Sinkala M., Kankasa C., Mwiya M., Thea D.M., Aldrovandi G.M. (2012). Human milk oligosaccharide concentration and risk of postnatal transmission of HIV through breastfeeding. Am. J. Clin. Nutr..

[9] Gao X., McMahon R.J., Woo J.G., Davidson B.S., Morrow A.L., Zhang Q. (2012). Temporal changes in milk proteomes reveal developing milk functions. J. Proteom. Res..

[10] Brandtzaeg P., Johansen F.E. (2007). IgA and intestinal homeostasis. Mucosal Immune Defense: Immunoglobulin A.

[11] Corthésy B. (2007). Roundtrip ticket for secretory IgA: Role in mucosal homeostasis?. J. Immunol..

[12] Newburg D.S., Walker W. (2007). Protection of the Neonate by the Innate Immune System of Developing Gut and of Human Milk. Pediatr. Res..

[13] Haneberg B. (1974). Immunoglobulins in feces from infants fed human or bovine milk. Scand. J. Immunol..

[14] Brandtzaeg P. (2003). Mucosal immunity: Integration between mother and the breast-fed infant. Vaccine.

[15] Demers-Mathieu V., Underwood M.A., Beverly R.L., Nielsen S.D., Dallas D.C. (2018). Comparison of human milk immunoglobulin survival during gastric digestion between preterm and term infants. Nutrients.

[16] Schlaudecker E.P., Steinhoff M.C., Omer S.B., McNeal M.M., Roy E., Arifeen S.E., Dodd C.N., Raqib R., Breiman R.F., Zaman K. (2013). IgA and neutralizing antibodies to influenza a virus in human milk: A randomized trial of antenatal influenza immunization. PLoS ONE.

[17] Fox A., Marino J., Amanat F., Krammer F., Hahn-Holbrook J., Zolla-Pazner S., Powell R.L. (2020). Robust and specific secretory IgA against SARS-CoV-2 detected in human milk. Iscience.

[18] Pace R.M., Williams J.E., Järvinen K.M., Belfort M.B., Pace C.D., Lackey K.A., Gogel A.C., Nguyen-Contant P., Kanagaiah P., Fitzgerald T. (2020). COVID-19 and human milk: SARS-CoV-2, antibodies, and neutralizing capacity. Medrxiv.

[19] Ohlsson A., Lacy J.B. (2013). Intravenous immunoglobulin for preventing infection in preterm and/or low birth weight infants. Cochrane Database Syst. Rev..

[20] Foster J.P., Seth R., Cole M.J. (2016). Oral immunoglobulin for preventing necrotizing enterocolitis in preterm and low birth weight neonates. Cochrane Database Syst. Rev..

[21] Lewis E.D., Richard C., Larsen B.M., Field C.J. (2017). The importance of human milk for immunity in preterm infants. Clin. Perinatol..

[22] Smilowitz J.T., Lebrilla C.B., Mills D.A., German J.B., Freeman S.L. (2014). Breast Milk Oligosaccharides: Structure-Function Relationships in the Neonate. Annu. Rev. Nutr..

[23] Azad M.B., Robertson B., Atakora F., Becker A.B., Subbarao P., Moraes T.J., Mandhane P.J., Turvey S.E., Lefebvre D.L., Sears M.R. (2018). Human Milk Oligosaccharide Concentrations Are Associated with Multiple Fixed and Modifiable Maternal Characteristics, Environmental Factors, and Feeding Practices. J. Nutr..

[24] Bode L. (2012). Human milk oligosaccharides: Every baby needs a sugar mama. Glycobiology.

[25] Albrecht S., Schols H., van den Heuvel E., Voragen A., Gruppen H. (2011). Occurrence of oligosaccharides in feces of breast-fed babies in their first six months of life and the corresponding breast milk. Carbohydr. Res..

[26] Wiciński M., Sawicka E., Gębalski J., Kubiak K., Malinowski B. (2020). Human milk oligosaccharides: Health benefits, potential applications in infant formulas, and pharmacology. Nutrients.

[27] Eiwegger T., Stahl B., Haidl P., Schmitt J., Boehm G., Dehlink E., Urbanek R., Szepfalusi Z. (2010). Prebiotic oligosaccharides: In vitro evidence for gastrointestinal epithelial transfer and immunomodulatory properties. Pediatr. Allergy Immunol..

[28] Morozov V., Hansman G., Hanisch F.G., Schroten H., Kunz C. (2018). Human milk oligosaccharides as promising antivirals. Mol. Nutr. Food Res..

[29] Guerrero C.A., Acosta O. (2016). Inflammatory and oxidative stress in rotavirus infection. World J. Virol..

[30] Etzold S., Bode L. (2014). Glycan-dependent viral infection in infants and the role of human milk oligosaccharides. Curr. Opin. Virol..

[31] Pannaraj P.S., Li F., Cerini C., Bender J.M., Yang S., Rollie A., Adisetiyo H., Zabih S., Lincez P.J., Bittinger K. (2017). Association between breast milk bacterial communities and establishment and development of the infant gut microbiome. JAMA Pediatr..

[32] Steenhout P., Sperisen P., Martin F.P., Sprenger N., Wernimont S., Pecquet S., Berger B. (2016). Term Infant Formula Supplemented with Human Milk Oligosaccharides (2′ Fucosyllactose and Lacto-N-neotetraose) Shifts Stool Microbiota and Metabolic Signatures Closer to that of Breastfed Infants. FASEB J..

[33] Roessler A., Friedrich U., Vogelsang H., Bauer A., Kaatz M., Hipler U.C., Schmidt I., Jahreis G. (2008). The immune system in healthy adults and patients with atopic dermatitis seems to be affected differently by a probiotic intervention. Clin. Exp. Allergy.

[34] Viljanen M., Kuitunen M., Haahtela T., Juntunen-Backman K., Korpela R., Savilahti E. (2005). Probiotic effects on faecal inflammatory markers and on faecal IgA in food allergic atopic eczema/dermatitis syndrome infants. Pediatr. Allergy Immunol..

[35] Donovan S.M., Comstock S.S. (2016). Human milk oligosaccharides influence neonatal mucosal and systemic immunity. Ann. Nutr. Metab..

[36] Parashar U.D., Hummelman E.G., Bresee J.S., Miller M.A., Glass R.I. (2003). Global illness and deaths caused by rotavirus disease in children. Emerg. Infect. Dis..

[37] López S., Arias C.F. (2004). Multistep entry of rotavirus into cells: A Versaillesque dance. Trends Microbiol..

[38] Charpilienne A., Abad M.J., Michelangeli F., Alvarado F., Vasseur M., Cohen J., Ruiz M.C. (1997). Solubilized and cleaved VP7, the outer glycoprotein of rotavirus, induces permeabilization of cell membrane vesicles. J. Gen. Virol..

[39] Chemello M.E., Aristimuño O.C., Michelangeli F., Ruiz M.C. (2002). Requirement for vacuolar H+-ATPase activity and Ca^2+^ gradient during entry of rotavirus into MA104 cells. J. Virol..

[40] Laucirica D.R., Triantis V., Schoemaker R., Estes M.K., Ramani S. (2017). Milk oligosaccharides inhibit human rotavirus infectivity in MA104 cells. J. Nutr..

[41] Ramani S., Stewart C.J., Laucirica D.R., Ajami N.J., Robertson B., Autran C.A., Shinge D., Rani S., Anandan S., Hu L. (2018). Human milk oligosaccharides, milk microbiome and infant gut microbiome modulate neonatal rotavirus infection. Nat. Commun..

[42] Chassaing M., Boudaud N., Belliot G., Estienney M., Majou D., de Rougemont A., Gantzer C. (2020). Interaction between norovirus and Histo-Blood Group Antigens: A key to understanding virus transmission and inactivation through treatments?. Food Microbiol..

[43] Tan M., Jiang X. (2011). Norovirus–host interaction: Multi-selections by human histo-blood group antigens. Trends Microbiol..

[44] Marionneau S., Ruvoën N., Le Moullac-Vaidye B., Clement M., Cailleau-Thomas A., Ruiz-Palacois G., Huang P., Jiang X., Le Pendu J. (2002). Norwalk virus binds to histo-blood group antigens present on gastroduodenal epithelial cells of secretor individuals. Gastroenterology.

[45] Schroten H., Hanisch F.G., Hansman G.S. (2016). Human norovirus interactions with histo-blood group antigens and human milk oligosaccharides. J. Virol..

[46] Hanisch F.G., Hansman G.S., Morozov V., Kunz C., Schroten H. (2018). Avidity of α-fucose on human milk oligosaccharides and blood group-unrelated oligo/polyfucoses is essential for potent norovirus-binding targets. J. Biol. Chem..

[47] Eldholm V., Rieux A., Monteserin J., Lopez J.M., Palmero D., Lopez B., Ritacco V., Didelot X., Balloux F. (2016). Impact of HIV co-infection on the evolution and transmission of multidrug-resistant tuberculosis. eLife.

[48] Little K.M., Kilmarx P.H., Taylor A.W., Rose C.E., Rivadeneira E.D., Nesheim S.R. (2012). A review of evidence for transmission of HIV from children to breastfeeding women and implications for prevention. Pediatr. Infect. Dis. J..

[49] Granelli-Piperno A., Pritsker A., Pack M., Shimeliovich I., Arrighi J.F., Park C.G., Trumpfheller C., Piguet V., Moran T.M., Steinman R.M. (2005). Dendritic cell-specific intercellular adhesion molecule 3-grabbing nonintegrin/CD209 is abundant on macrophages in the normal human lymph node and is not required for dendritic cell stimulation of the mixed leukocyte reaction. J. Immunol..

[50] Woodham A.W., Skeate J.G., Sanna A.M., Taylor J.R., Da Silva D.M., Cannon P.M., Kast W.M. (2016). Human Immunodeficiency Virus Immune Cell Receptors, Coreceptors, and Cofactors: Implications for Prevention and Treatment. AIDS Patient Care STDs.

[51] Hong P., Ninonuevo M.R., Lee B., Lebrilla C., Bode L. (2008). Human milk oligosaccharides reduce HIV-1-gp120 binding to dendritic cell-specific ICAM3-grabbing non-integrin (DC-SIGN). Br. J. Nutr..

[52] Van Niekerk E., Autran C.A., Nel D.G., Kirsten G.F., Blaauw R., Bode L. (2014). Human milk oligosaccharides differ between HIV-infected and HIV-uninfected mothers and are related to necrotizing enterocolitis incidence in their preterm very-low-birth-weight infants. J. Nutr..

[53] Rai D., Adelman A.S., Zhuang W., Rai G.P., Boettcher J., Lönnerdal B. (2014). Longitudinal changes in lactoferrin concentrations in human milk: A global systematic review. Crit. Rev. Food Sci. Nutr..

[54] Perrin M.T., Fogleman A.D., Newburg D.S., Allen J.C. (2017). A longitudinal study of human milk composition in the second year postpartum: Implications for human milk banking. Matern. Child Nutr..

[55] Demmelmair H., Prell C., Timby N., Lönnerdal B. (2017). Benefits of Lactoferrin, Osteopontin and Milk Fat Globule Membranes for Infants. Nutrients.

[56] Suzuki Y.A., Lopez V., Lönnerdal B. (2005). Lactoferrin. Cell. Mol. Life Sci..

[57] Legrand D. (2016). Overview of lactoferrin as a natural immune modulator. J. Pediatr..

[58] Van der Strate B.W.A., Beljaars L., Molema G., Harmsen M.C., Meijer D.K.F. (2001). Antiviral activities of lactoferrin. Antivir. Res..

[59] Redwan E.M., Uversky V.N., El-Fakharany E.M., Al-Mehdar H. (2014). Potential lactoferrin activity against pathogenic viruses. C. R. Biol..

[60] Hasegawa K., Motsuchi W., Tanaka S., Dosako S.I. (1994). Inhibition with lactoferrin of in vitro infection with human herpes virus. Jpn. J. Med. Sci. Biol..

[61] Andersen J.H., Osbakk S.A., Vorland L.H., Traavik T., Gutteberg T.J. (2001). Lactoferrin and cyclic lactoferricin inhibit the entry of human cytomegalovirus into human fibroblasts. Antivir. Res..

[62] Hammer J., Haaheim H., Gutteberg T.J. (2000). Bovine lactoferrin is more efficient than bovine lactoferricin in inhibiting HSV-I/-II replication in vitro. Lactoferrin: Structure, Function and Applications, Proceedings of the 4th International Conference on Lactoferrin: Structure, Function and Applications, Sapporo, Japan, 18–22 May 1999.

[63] Marchetti M., Pisani S., Antonini G., Valenti P., Seganti L., Orsi N. (1998). Metal complexes of bovine lactoferrin inhibit in vitro replication of herpes simplex virus type 1 and 2. Biometals.

[64] Swart P.J., Kuipers E.M., Smit C., Van Der Strate B.W., Harmsen M.C., Meijer D.K. (1998). Lactoferrin. Advances in Lactoferrin Research.

[65] Weimer K.E., Roark H., Fisher K., Cotten C.M., Kaufman D.A., Bidegain M., Permar S.R. (2020). Breast milk and saliva lactoferrin levels and postnatal cytomegalovirus infection. Am. J. Perinatol..

[66] Damiens E., El Yazidi I., Mazurier J., Elass-Rochard E., Duthille I., Spik G., Boilly-Marer Y. (1998). Role of heparan sulphate proteoglycans in the regulation of human lactoferrin binding and activity in the MDA-MB-231 breast cancer cell line. Eur. J. Cell Biol..

[67] Sarrazin S., Lamanna W.C., Esko J.D. (2011). Heparin sulphate proteoglycans. Cold Spring Harbor Perspect. Biol..

[68] Kell D.B., Heyden E.L., Pretorius E. (2020). The Biology of Lactoferrin, an Iron-Binding Protein that Can Help Defend Against Viruses and Bacteria. Front. Immunol..

[69] Lang J., Yang N., Deng J., Liu K., Yang P., Zhang G., Jiang C. (2011). Inhibition of SARS pseudovirus cell entry by lactoferrin binding to heparan sulfate proteoglycans. PLoS ONE.

[70] Mulder A.M., Connellan P.A., Oliver C.J., Morris C.A., Stevenson L.M. (2008). Bovine lactoferrin supplementation supports immune and antioxidant status in healthy human males. Nutr. Res..

[71] Griffiths J., Jenkins P., Vargova M., Bowler U., Juszczak E., King A., Linsell L., Murray D., Partlett C., Patel M. (2019). Enteral lactoferrin supplementation for very preterm infants: A randomised placebo-controlled trial. Lancet.

[72] Nwosu C.C., Aldredge D.L., Lee H., Lerno L.A., Zivkovic A.M., German J.B., Lebrilla C.B. (2012). Comparison of the human and bovine milk N-glycome via high-performance microfluidic chip liquid chromatography and tandem mass spectrometry. J. Proteom Res..

[73] Doyle L.W., Cheong J.L. (2019). Does bovine lactoferrin prevent late-onset neonatal sepsis?. Lancet.

[74] Ibrahim H.R., Imazato K., Ono H. (2011). Human lysozyme possesses novel antimicrobial peptides within its N-terminal domain that target bacterial respiration. J. Agric. Food Chem..

[75] Ogundele M.O. (1999). Inhibitors of complement activity in human breast-milk: A proposed hypothesis of their physiological significance. Mediat. Inflamm..

[76] Behbahani M., Nosrati M., Mohabatkar H. (2018). Inhibition of human immunodeficiency type 1 virus (HIV-1) life cycle by different egg white lysozymes. Appl. Biochem. Biotechnol..

[77] Lee-Huang S., Maiorov V., Huang P.L., Ng A., Lee H.C., Chang Y.T., Kallenbach N., Huang P.L., Chen H.C. (2005). Structural and functional modeling of human lysozyme reveals a unique nonapeptide, HL9, with anti-HIV activity. Biochemistry.

[78] Ella E.E., Ahmad A.A., Umoh V.J., Ogala W.N., Balogun T.B., Musa A. (2011). Studies on the interaction between IgA, lactoferrin and lysozyme in the breastmilk of lactating women with sick and healthy babies. J. Infect. Dis. Immun..

[79] Montagne P., Cuilliere M.L., Mole C., Bene M.C., Faure G. (2001). Changes in lactoferrin and lysozyme levels in human milk during the first twelve weeks of lactation. Bioactive Components of Human Milk.

[80] Newburg D.S., Peterson J.A., Ruiz-Palacios G.M., Matson D.O., Morrow A.L., Shults J., de Lourdes Guerrero M., Chaturvedi P., Newburg S.O., Scallan C.D. (1998). Role of human-milk lactadherin in protectoin against symptomatic rotavirus infection. Lancet.

[81] He Y., Lawlor N.T., Newburg D.S. (2016). Human Milk Components Modulate Toll-Like Receptor-Mediated Inflammation. Adv. Nutr..

[82] Yolken R.H., Peterson J.A., Vonderfecht S.L., Fouts E.T., Midthun K., Newburg D.S. (1992). Human milk mucin inhibits rotavirus replication and prevents experimental gastroenteritis. J. Clin. Investig..

[83] Shen B., Mei M., Pu Y., Zhang H., Liu H., Tang M., Pan Q., He Y., Wu X., Zhao H. (2019). Necrostatin-1 attenuates renal ischemia and reperfusion injury via meditation of HIF-1α/mir-26a/TRPC6/PARP1 signaling. Mol. Ther. Nucleic Acids.

[84] Midwood K.S., Hussenet T., Langlois B., Orend G. (2011). Advances in tenascin-C biology. Cell. Mol. Life Sci..

[85] Fouda G.G., Jaeger F.H., Amos J.D., Ho C., Kunz E.L., Anasti K., Stamper L.W., Liebl B.E., Barbas K.H., Ohashi T. (2013). Tenascin-C is an innate broad-spectrum, HIV-1–neutralizing protein in breast milk. Proc. Natl. Acad. Sci. USA.

[86] Mansour R.G., Stamper L., Jaeger F., McGuire E., Fouda G., Amos J., Barbas K., Ohashi T., Alam S.M., Erickson H. (2016). The Presence and Anti-HIV-1 Function of Tenascin C in Breast Milk and Genital Fluids. PLoS ONE.

[87] Mangan R.J., Stamper L., Ohashi T., Eudailey J.A., Go E.P., Jaeger F.H., Itell H.L., Watts B.E., Fouda G.G., Erickson H.P. (2019). Determinants of Tenascin-C and HIV-1 envelope binding and neutralization. Mucosal Immunol..

[88] Karlsson O., Rodosthenous R.S., Jara C., Brennan K.J., Wright R.O., Baccarelli A.A., Wright R.J. (2016). Detection of long non-coding RNAs in human breastmilk extracellular vesicles: Implications for early child development. Epigenetics.

[89] Zempleni J., Aguilar-Lozano A., Sadri M., Sukreet S., Manca S., Wu D., Zhou F., Mutai E. (2017). Biological Activities of Extracellular Vesicles and Their Cargos from Bovine and Human Milk in Humans and Implications for Infants. J. Nutr..

[90] Van Herwijnen M.J., Zonneveld M.I., Goerdayal S., Nolte E.N., Garssen J., Stahl B., Altelaar A.M., Redegeld F.A., Wauben M.H. (2016). Comprehensive proteomic analysis of human milk-derived extracellular vesicles unveils a novel functional proteome distinct from other milk components. Mol. Cell. Proteom..

[91] Admyre C., Johansson S.M., Qazi K.R. (2007). Exosomes with immune modulatory features are present in human breast milk. J. Immunol..

[92] Sinha A., Maurice C.F. (2019). Bacteriophages: Uncharacterized and dynamic regulators of the immune system. Mediat. Inflamm..

[93] Breitbart M., Haynes M., Kelley S., Angly F., Edwards R.A., Felts B., Mahaffy J.M., Mueller J., Nulton J., Rayhawk S. (2008). Viral diversity and dynamics in an infant gut. Res. Microbiol..

[94] Duranti S., Lugli G.A., Mancabelli L., Armanini F., Turroni F., James K., Ferretti P., Gorfer V., Ferrario C., Milani C. (2017). Maternal inheritance of bifidobacterial communities and bifidophages in infants through vertical transmission. Microbiome.

[95] Pannaraj P.S., Ly M., Cerini C., Saavedra M., Aldrovandi G.M., Saboory A.A., Johnson K.M., Pride D.T. (2018). Shared and distinct features of human milk and infant stool viromes. Front. Microbiol..

[96] Manrique P., Bolduc B., Walk S.T., van der Oost J., de Vos W.M., Young M.J. (2016). Healthy human gut phageome. Proc. Natl. Acad. Sci. USA.

[97] Liang G., Zhao C., Zhang H., Mattei L., Sherrill-Mix S., Bittinger K., Kessler L.R., Wu G.D., Baldassano R.N., DeRusso P. (2020). The stepwise assembly of the neonatal virome is modulated by breastfeeding. Nature.

[98] Ma Y., You X., Mai G., Tokuyasu T., Liu C. (2018). A human gut phage catalog correlates the gut phageome with type 2 diabetes. Microbiome.

[99] Norman J.M., Handley S.A., Baldridge M.T., Droit L., Liu C.Y., Keller B.C., Kambal A., Monaco C.L., Zhao G., Fleshner P. (2015). Disease-specific alterations in the enteric virome in inflammatory bowel disease. Cell.

[100] Brenmoehl J., Ohde D., Wirthgen E., Hoeflich A. (2018). Cytokines in milk and the role of TGF-beta. Best Pract. Res. Clin. Endocrinol. Metab..

[101] Field C.J. (2005). The immunological components of human milk and their effect on immune development in infants. J. Nutr..

[102] Garofalo R. (2010). Cytokines in human milk. J. Pediatr..

[103] Saito S., Yoshida M., Ichijo M., Ishizaka S., Tsujh T. (1993). Transforming growth factor-beta (TGF-β) in human milk. Clin. Exp. Immunol..

[104] Nakamura Y., Miyata M., Ando T., Shimokawa N., Ohnuma Y., Katoh R., Ogawa H., Okumura K., Nakao A. (2009). The latent form of transforming growth factor-b administered orally is activated by gastric acid in mice. J. Nutr..

[105] Lee S.H. (2015). Intestinal permeability regulation by tight junction: Implication on inflammatory bowel diseases. Intestinal Res..

[106] Donnet-Hughes A., Duc N., Serrant P., Vidal K., Schiffrin E. (2000). Bioactive molecules in milk and their role in health and disease: The role of transforming growth factor-β. Immunol. Cell Biol..

[107] Maheshwari A., Kelly D.R., Nicola T., Ambalavanan N., Jain S.K., Murphy–Ullrich J., Athar M., Shimamura M., Bhandari V., Aprahamian C. (2011). TGF-β2 suppresses macrophage cytokine production and mucosal inflammatory responses in the developing intestine. Gastroenterology.

[108] Siggers R.H., Siggers J., Boye M., Thymann T., Mølbak L., Leser T., Jensen B.B., Sangild P.T. (2008). Early administration of probiotics alters bacterial colonization and limits diet-induced gut dysfunction and severity of necrotizing enterocolitis in preterm pigs. J. Nutr..

[109] Howe K.L., Reardon C., Wang A., Nazli A., McKay D.M. (2005). Transforming growth factor-β regulation of epithelial tight junction proteins enhances barrier function and blocks enterohemorrhagic Escherichia coli O157:H7-induced increased permeability. Am. J. Pathol..

[110] Srivastava M.D., Srivastava A., Brouhard B., Saneto R., Groh-Wargo S., Kubit J. (1996). Cytokines in human milk. Res. Commun. Mol. Pathol. Pharmacol..

[111] Coppa G.V., Gabrielli O., Buzzega D., Zampini L., Galeazzi T., Maccari F., Bertino E., Volpi N. (2011). Composition and structure elucidation of human milk glycosaminoglycans. Glycobiology.

[112] Coppa G.V., Gabrielli O., Bertino E., Zampini L., Galeazzi T., Padella L., Santoro L., Marchesiello R.L., Galeotti F., Maccari F. (2013). Human milk glycosaminoglycans: The state of the art and future perspectives. Ital. J. Pediatr..

[113] Raman R., Sasisekharan V., Sasisekharan R. (2005). Structural insights into biological roles of protein-glycosaminoglycan interactions. Chem. Biol..

[114] Coppa G.V., Gabrielli O., Zampini L., Galeazzi T., Maccari F., Buzzega D., Galeotti F., Bertino E., Volpi N. (2012). Glycosaminoglycan Content in Term and Preterm Milk during the First Month of Lactation. Neonatology.

[115] Newburg D.S., Linhardt R.J., Ampofo S.A., Yolken R.H. (1995). Human Milk Glycosaminoglycans Inhibit HIV Glycoprotein gp120 Binding to its Host Cell CD4 Receptor. J. Nutr..

[116] Egea J., García A.G., Verges J., Montell E., López M.G. (2010). Antioxidant, antiinflammatory and neuroprotective actions of chondroitin sulfate and proteoglycans. Osteoarthr. Cartilage.

[117] Rossi S., Bonferoni M.C., Ferrari F., Bertoni M., Caramella C. (1996). Characterization of mucin interaction with three viscosity grades of sodium carboxymethylcellulose. Comparison between rheological and tensile testing. Eur. J. Pharm. Sci..

[118] Zhang J., Perez A., Yasin M., Soto P., Rong M., Theodoropoulos G., Carothers Carraway C.A., Carraway K.L. (2005). Presence of MUC4 in human milk and at the luminal surfaces of blood vessels. J. Cell. Physiol..

[119] Mall A.S., Habte H., Mthembu Y., Peacocke J., De Beer C. (2017). Mucus and Mucins: Do they have a role in the inhibition of the human immunodeficiency virus?. Virology.

[120] Mthembu Y., Lotz Z., Tyler M., de Beer C., Rodrigues J., Schoeman L., Mall A.S. (2014). Purified human breast milk MUC1 and MUC4 inhibit human immunodeficiency virus. Neonatology.

[121] Habte H.H., De Beer C., Lotz Z.E., Tyler M.G., Kahn D., Mall A.S. (2008). Inhibition of human immunodeficiency virus type 1 activity by purified human breast milk mucin (MUC1) in an inhibition assay. Neonatology.

[122] Kazmi S.H., Naglik N.J.R., Sweet S.P., Evans R.W., O’Shea S., Banatvala J.E., Challacombe S.J. (2006). Comparison of human immunodeficiency virus type 1-specific inhibitory activities in saliva and other human mucosal fluids. Clin. Vaccine Immunol..

[123] Foxall C., Watson S.R., Dowbenko D., Fennie C., Lasky L.A., Kiso M., Hasegawa A., Asa D., Brandley B.K. (1992). The three members of the selectin receptor family recognize a common carbohydrate epitope, the sialyl Lewis (x) oligosaccharide. J. Cell Biol..

[124] Naarding M.A., Ludwig I.S., Groot F., Berkhout B., Geijtenbeek T.B., Pollakis G., Paxton W.A. (2005). Lewis X component in human milk binds DC-SIGN and inhibits HIV-1 transfer to CD4+ T lymphocytes. J. Clin. Investig..

[125] Lyimo M.A., Mosi M.N., Housman M.L., Zain-Ul-Abideen M., Lee F.V., Howell A.L., Connor R.I. (2012). Breast milk from Tanzanian women has divergent effects on cell-free and cell-associated HIV-1 infection in vitro. PLoS ONE.

[126] Koning N., Kessen S.F., Van Der Voorn J.P., Appelmelk B.J., Jeurink P.V., Knippels L.M., Garssen J., Van Kooyk Y. (2015). Human Milk Blocks DC-SIGN-Pathogen Interaction via MUC1. Front. Immunol..

[127] Enig M.G. Coconut: In support of good health in the 21st century. Proceedings of the 36th Meeting of APCC.

[128] Nduati R.W., John G.C., Richardson B.A., Overbaugh J., Welch M., Ndinya-Achola J., Moses S., Holmes K., Onyango F., Kreiss J.K. (1995). Human immunodeficiency virus type 1-infected cells in breast milk: Association with immunosuppression and vitamin A deficiency. J. Infect. Dis..

[129] Fawzi W.W., Msamanga G.I., Hunter D., Renjifo B., Antelman G., Bang H., Manji K., Kapiga S., Mwakagile D., Essex M. (2002). Randomized trial of vitamin supplements in relation to transmission of HIV-1 through breastfeeding and early child mortality. Aids.

[130] Russell R.M. (2000). The vitamin A spectrum: From deficiency to toxicity. Am. J. Clin. Nutr..

[131] Ahmad S.M., Hossain M.I., Bergman P., Kabir Y., Raqib R. (2015). The effect of postpartum vitamin A supplementation on breast milk immune regulators and infant immune functions: Study protocol of a randomized, controlled trial. Trials.

[132] Rueda R. (2013). 13—Gangliosides, immunity, infection and inflammation. Woodhead Publishing Series in Food Science, Technology and Nutrition, Diet, Immunity and Inflammation.

[133] Takamizawa K., Iwamori M., Mutai M., Nagai Y. (1986). Gangliosides of bovine buttermilk. Isolation and characterization of a novel monosialoganglioside with a new branching structure. J. Biol. Chem..

[134] Schnabl K.L., Larsen B., Van Aerde J.E., Lees G., Evans M., Belosevic M., Field C., Thomson A.B.R., Clandinin M.T. (2009). Gangliosides protect bowel in an infant model of necrotizing enterocolitis by suppressing proinflammatory signals. J. Pediatr. Gastroenterol. Nutr..

[135] Sasarman F., Maftei C., Campeau P.M., Brunel-Guitton C., Mitchell G.A., Allard P. (2016). Biosynthesis of glycosaminoglycans: Associated disorders and biochemical tests. J. Inherit. Metab. Dis..

[136] Burge K.Y., Hannah L., Eckert J.V., Gunasekaran A., Chaaban H. (2019). The protective influence of chondroitin sulfate, a component of human milk, on intestinal bacterial invasion and translocation. J. Hum. Lact..

[137] Liu S.Y., Aliyari R., Chikere K., Li G., Marsden M.D., Smith J.K., Pernet O., Guo H., Nusbaum R., Zack J.A. (2013). Interferon-inducible cholesterol-25-hydroxylase broadly inhibits viral entry by production of 25-hydroxycholesterol. Immunity.

[138] Cagno V., Civra A., Rossin D., Calfapietra S., Caccia C., Leoni V., Dorma N., Biasi F., Poli G., Lembo D. (2017). Inhibition of herpes simplex-1 virus replication by 25-hydroxycholesterol and 27-hydroxycholesterol. Redox Biol..

[139] Civra A., Francese R., Gamba P., Testa G., Cagno V., Poli G., Lembo D. (2018). 25-Hydroxycholesterol and 27-hydroxycholesterol inhibit human rotavirus infection by sequestering viral particles into late endosomes. Redox Biol..

[140] Civra A., Leoni V., Caccia C., Sottemano S., Tonetto P., Coscia A., Peila C., Moro G.E., Gaglioti P., Bertino E. (2019). Antiviral oxysterols are present in human milk at diverse stages of lactation. J. Steroid Biochem. Mol. Biol..

[141] Fan H., Hong B., Luo Y., Peng Q., Wang L., Jin X., Chen Y., Hu Y., Shi Y., Li T. (2020). The effect of whey protein on viral infection and replication of SARS-CoV-2 and pangolin coronavirus in vitro. Signal Transduct. Target. Ther..

